# Non-destructive Determination of Shikimic Acid Concentration in Transgenic Maize Exhibiting Glyphosate Tolerance Using Chlorophyll Fluorescence and Hyperspectral Imaging

**DOI:** 10.3389/fpls.2018.00468

**Published:** 2018-04-09

**Authors:** Xuping Feng, Chenliang Yu, Yue Chen, Jiyun Peng, Lanhan Ye, Tingting Shen, Haiyong Wen, Yong He

**Affiliations:** ^1^College of Biosystems Engineering and Food Science, Zhejiang University, Hangzhou, China; ^2^Key Laboratory of Spectroscopy, Ministry of Agriculture, Hangzhou, China; ^3^Vegetable Research Institute, Zhejiang Academy of Agricultural Sciences, Hangzhou, China; ^4^Institute of Horticulture, Zhejiang Academy of Agricultural Sciences, Hangzhou, China

**Keywords:** chemometric analysis, chlorophyll fluorescence imaging, hyperspectral imaging, plant breeding, transgenic maize

## Abstract

The development of transgenic glyphosate-tolerant crops has revolutionized weed control in crops in many regions of the world. The early, non-destructive identification of superior plant phenotypes is an important stage in plant breeding programs. Here, glyphosate-tolerant transgenic maize and its parental wild-type control were studied at 2, 4, 6, and 8 days after glyphosate treatment. Visible and near-infrared hyperspectral imaging and chlorophyll fluorescence imaging techniques were applied to monitor the performance of plants. In our research, transgenic maize, which was highly tolerant to glyphosate, was phenotyped using these high-throughput non-destructive methods to validate low levels of shikimic acid accumulation and high photochemical efficiency of photosystem II as reflected by maximum quantum yield and non-photochemical quenching in response to glyphosate. For hyperspectral imaging analysis, the combination of spectroscopy and chemometric methods was used to predict shikimic acid concentration. Our results indicated that a partial least-squares regression model, built on optimal wavelengths, effectively predicted shikimic acid concentrations, with a coefficient of determination value of 0.79 for the calibration set, and 0.82 for the prediction set. Moreover, shikimic acid concentration estimates from hyperspectral images were visualized on the prediction maps by spectral features, which could help in developing a simple multispectral imaging instrument for non-destructive phenotyping. Specific physiological effects of glyphosate affected the photochemical processes of maize, which induced substantial changes in chlorophyll fluorescence characteristics. A new data-driven method, combining mean fluorescence parameters and featuring a screening approach, provided a satisfactory relationship between fluorescence parameters and shikimic acid content. The glyphosate-tolerant transgenic plants can be identified with the developed discrimination model established on important wavelengths or sensitive fluorescence parameters 6 days after glyphosate treatment. The overall results indicated that both hyperspectral imaging and chlorophyll fluorescence imaging techniques could provide useful tools for stress phenotyping in maize breeding programs and could enable the detection and evaluation of superior genotypes, such as glyphosate tolerance, with a non-destructive high-throughput technique.

## Introduction

Weed management in maize production is essential to maximize yield and to achieve a good harvest. Glyphosate is a broad-spectrum herbicide possessing several desirable characteristics, such as environmental safety, broad-spectrum mode-of-action, and low cost (Sammons and Gaines, 2014). The development of transgenic glyphosate-tolerant crop cultivars has allowed this non-selective herbicide to be used for post-emergence weed control in crops. Glyphosate is the only commercial herbicide which operates by blocking the activity of the enzyme 5-enolpyruvylshikimate-3-phosphate synthase (EPSPS) (Sammons and Gaines, 2014). Extensive research has illustrated that tolerance of high concentrations of glyphosate depends on the expression of an EPSPS with a low binding affinity for glyphosate (Duke et al., 2017). Genetic engineering of the *cp4-epsps* gene from *Agrobacterium tumefaciens* strain CP4 into maize and other crops achieved glyphosate-specific herbicide tolerance and subsequently improved the control of weeds in these transgenic crop cultivars (Howe et al., 2002).

An important step in plant breeding is selection (usually more accurately described as “screening”), by which superior plant phenotypes are identified for the development of improved cultivars better suited to the needs of farmers and consumers. Visual phenotypic selection can be slow and inefficient, particularly with respect to quantitative traits. Glyphosate is a relatively slow-acting herbicide, and visual assessment of herbicide injury may take several weeks (Singh and Shaner, 1998). An early and non-destructive assessment of glyphosate phytotoxicity damage would be useful for identifying glyphosate-tolerant individuals in a segregating breeding population. Previous research had demonstrated that the lack of shikimate accumulation in glyphosate-treated plants could be used as an indicator of glyphosate resistance (Padgette et al., 1995; Mueller et al., 2003). Standard biochemical detection techniques, such as spectrophotometry or high-performance liquid chromatography, have been used to quantify shikimic acid concentration in glyphosate-treated plant tissues (Pline et al., 2002). These methods yield accurate results, but they are time consuming, labor intensive and destructive, and cannot meet the needs of large-scale screening programs. Maize breeding programs need a quick and easy tool for assessing phenotypes, which allows the non-destructive screening of large numbers of plants, making it possible to identify desired individuals early in the screening process. Therefore, more rapid and less expensive alternative high-throughput approaches are needed for the screening and identification of glyphosate-tolerant maize plants.

Hyperspectral imaging (HSI) integrates spectroscopy and imaging techniques, and has been employed as a non-invasive imaging technology for the evaluation of quantitative and qualitative changes caused by abiotic or biotic stresses at the leaf and canopy levels (Li et al., 2014; Lowe et al., 2017). For plant stress, the most commonly used wavelengths are in the visible region (400–700 nm) to observe photoactive pigments, the near-infrared region (750–1200 nm) to detect leaf water content and mesophyll cell structure, and the shortwave infrared region (1200–2400 nm) to investigate biochemical components (Lowe et al., 2017). Recently, numbers of reviews have been published on the potential applications and advantages of high-throughput plant screening phenotyping methods based on HIS (Li et al., 2014; Humplík et al., 2015; Mutka and Bart, 2015; Walter et al., 2015). This technique has been successfully using in plant phonemics applications to estimate plant variation in response to stresses caused by salt (Sytar et al., 2017), toxic metals (Rathod et al., 2013), disease (Ashourloo et al., 2014), drought and heat (Estrada et al., 2015), particularly at the early stages shortly after treatment (Lowe et al., 2017). However, the large size data generated from HSI often complicates the computing process and limits its high-throughput applications (Sun et al., 2015). In recent years, vegetative indicators that calculated as ratios or linear combinations with several spectral bands are used for plant trait detection and phenotyping. Such indices are, for example, normalized difference vegetation index, a bio-indicators for chlorophyll and other pigments, and the photochemical reflectance index, an estimator of the photosynthetic efficiency (Rathod et al., 2013). These vegetation indices have been used to predict the biomass, chlorophyll content, and yield for different plant (Li et al., 2014; Mutka and Bart, 2015; Walter et al., 2015). Glyphosate promotes deregulation of the shikimate pathway and can affect a series of events, such as blocking photosynthetic electron transport, inhibiting CO_2_ assimilation processes, and reducing chlorophyll and leaf water concentrations, which are ultimately reflections of biochemical, physiological, and cell structural changes in response to glyphosate exposure (Silva et al., 2014). There have been no studies reported on the application of HSI for high-throughput plant stress phenotyping to evaluate glyphosate tolerance in a plant breeding program. At more complex level, entire spectra should be analyzed combined with chemometric approaches that allow for comparison many wavelengths.

Chlorophyll fluorescence (ChlF) has been widely and successfully used as a tool for estimating plant physiological response to abiotic and biotic stresses (Porcarcastell et al., 2014), and has the potential to overcome the traditional screening and phenotyping limitations for glyphosate tolerance. This system not only measures the visible effects of stress-induced chlorophyll breakdown but also provides a comprehensive insight into the potential and actual efficiency of photosynthesis (Lichtenthaler and Miehé, 1997). ChlF imaging can be used to screen many plants simultaneously by providing information on both the spatial and temporal dynamics of photosynthesis (Porcarcastell et al., 2014). Moreover, ChlF is extremely sensitive to different environmental changes that affect the physiological process, such as herbicide stress (Barbagallo et al., 2003), toxic metals (Joshi and Mohanty, 2004), drought (Estrada et al., 2015), nutritional deficiencies (Kalaji et al., 2014) or disease (Cen et al., 2017). ChlF has been widely used in plant breeding as a basis for screening (Humplík et al., 2015; Justine et al., 2015; Awlia et al., 2016; Kalaji et al., 2016). Herbicides have many different modes of action to kill or inhibit the growth of target plant, one of which is inhibiting photosynthesis, such as diuron, isoproturon (Haynes et al., 2000; Laviale et al., 2011). Glyphosate, which inhibits the shikimate pathway, not only decreases the biosynthesis of aromatic amino acids, but also influences photosynthetic activity and induces substantial changes in chlorophyll fluorescence characteristics (Pavlović et al., 2014). It is possible that ChlF could achieve *in vivo* vitality screening, either to analyze the glyphosate effect on the photosynthetic physiological processes or to screen for individual plants of interest.

The aim of this study was to apply the non-destructive techniques HSI and ChlF imaging to evaluate differences in glyphosate tolerance between the transgenic (TG) maize and its parental wild-type (WT). We assumed that the specific physiological effects of glyphosate would affect photochemical processes captured by ChlF parameters, and induce physiological and biochemical changes detected by spectral information. Additional aims were: (1) to identify sensitive wavelengths from hyperspectral data and to establish a mathematical model for predicting shikimic acid concentration; (2) to discover the appropriate ChlF indicators for rapid detection of glyphosate-induced metabolic perturbations and to investigate the relationship between glyphosate-induced ChlF characteristics and the level of glyphosate stress; (3) to find the optimal imaging technique for high-throughput screening and characterization of the glyphosate-tolerant maize genotype to facilitate plant breeding programs.

## Materials and Methods

### Plant Material and Experimental Design

Seeds of transgenic glyphosate-tolerant (TG) maize and the corresponding wild-type (WT) line were provided by the Institute of Insect Sciences, Zhejiang University, Hangzhou, China. The glyphosate-tolerant maize line, SK12-5 (TG), was obtained after transformation of the WT (zhengdan958) with a gene encoding a glyphosate-insensitive 5-enolpyruvylshikimate-3-phosphate synthase (EPSPS) from *Agrobacterium* sp.

The experiments were conducted in a greenhouse at Zhejiang University, in October 2016. The average temperature registered in the greenhouse during the experimental period for 24-h was 20°C and the average relative humidity was 65%. Maize seeds, one per pot were sown in a plastic bucket (108 mm bottom diameter × 131 mm height) with drainage holes in a 1:1:1 mix of soil: calcined clay: torpedo sand. The soil used in the experiments was air-dried, and debris, weeds and gravel were removed before use.

On reaching the 3-leaf stage (second leaf fully expanded, third leaf emerging), plants were sprayed with either water or 1.08 kg a.i ha^-1^ glyphosate. A commercial formulation of glyphosate (Zhejiang Wynca Chemical Group Co., Ltd., Hangzhou, China) was dissolved in water. The samples that were treated with water were regarded to be the control. The treatments (glyphosate or water) were carried out, using a CO_2_ pressurized portable sprayer equipped with flat-fan nozzles that sprayed over a 0.7 m width from a distance of 1.5 m. Glyphosate efficacy was assessed 2, 4, 6, and 8 days after treatment, with 10 replicate pots of each control treatment and 20 replicate pots of each glyphosate treatment. In total, 120 plants of each of the TG and WT maize treatments were collected. For each collected days, there were 10 plants sprayed with water and 20 plants sprayed with glyphosate for TG or WT. The samples were collected for recording hyperspectral imaging and chlorophyll fluorescence imaging data during the experiment. After acquiring the hyperspectral imaging and chlorophyll fluorescence imaging data, the above-ground part of the maize plants were harvested to measure the shikimic acid concentration and chlorophyll content.

### Hyperspectral Imaging Acquisition

Hyperspectral images were acquired over the visible and near-infrared (Vis-NIR) range from 380 to 1030 nm using the following devices: an imaging spectrograph (ImSpector V10E; Spectral Imaging Ltd., Oulu, Finland), a high-performance charge-coupled device (CCD) camera [672 × 512 (spatial × spectral) pixels with a spectral resolution of 2.8 nm; Hamamatsu Photonics K.K., Hamamatsu City, Japan], coupled with a camera lens (OLES23; Spectral Imaging Ltd., Oulu, Finland), and two 150 W tungsten halogen lamps (9596ER; Dolan-Jenner Industries Inc., Boxborough, MA, United States) for illumination. The intact plant was placed on the conveyer belt to conduct linear HSI scanning. For image acquisition, the distance between the lens of the CCD camera and the plant canopy, the exposure time of the camera, and the speed of the conveyer belt were adjusted to 56.3 cm, 4 ms and 7 mm/s, respectively, before image acquisition to acquire clear and non-deformed images.

The original hyperspectral images were corrected to the dark and white reference images. The entire plant image was isolated from the soil and conveyer belt backgrounds, using the image segmentation method, and identified as the region of interest (ROI). For each ROI, the spectral reflectance values of all pixels within the area were averaged. Wavelet transformation, employing Daubechies 6 with decomposition scale 3 was used for removing the background in the spectrum (Carlomagno et al., 2004; Shao and Zhuang, 2004). Other preprocessing methods, namely, standard normal variate, multiplicative scatter correction and Savitzky–Golay smoothing were then implemented to decrease the noise and enhance possible spectral features related to the property studied. The spectral data preprocessing methods were compared and tested, but these preprocessing methods did not improve the predictive capability of the model, compared to the wavelet transformation procedures (Supplementary Table S1).

### Chlorophyll Fluorescence Imaging

ChlF images of the whole plants were captured at room temperature using a FluorCam 800 imaging system (Photon Systems Instruments, Brno, Czechia). The Chl-F emission transients were captured by a CCD camera (SV-H 1.4/6, VS Technology, Tokyo, Japan) in a series of images (696 pixels × 520 pixels, spectral × spatial). The system included four LED panels to supply actinic light and saturating flashes. The kinetic chlorophyll fluorescence imaging system was that previously described by Cen et al. (2017). The plant was dark adapted for 30 min before measurements were taken. *F*_m_ was recorded following a strong light flash (1,400 μmol photons m^-2^ s^-1^) at 5.56 s. Then, the plant was irradiated with continuous actinic light (120 μmol photons m^-2^ s^-1^) for 70 s, supplemented with five saturating pulses to measure the *F*_m_ signals during light adaptation (*F_m__Ln*) and steady-state *F*_m_ in light (*F_m__Lss*) at 31.92, 41.92, 51.92, 71.92, and 91.92 s of actinic light. Three saturated pulses were applied after switching off the actinic light to measure the *F*_m_ signals during dark relaxation (*F_m__Dn*) (Supplementary Figure S1). With this system, various fluorescence signals could be obtained. For each ROI, the fluorescence parameter values of all pixels within the area were averaged. Many different fluorescence parameters were used to characterize the various aspects of photosynthetic performance comprehensively.

The FluorCam7 (PSI) software allows the determination of the area generating a fluorescence signal in any given image. For maize plants, the area of the entire plant was estimated using ROI pixel numbers, and was used to monitor plant growth. ChlF transients of the entire plant imaged with the time points were analyzed. Once chlorophyll fluorescence images were obtained, the entire illuminated plant was freeze-clamped in liquid N_2_ and stored at -80°C until used for shikimic acid and chlorophyll concentration assays.

### Analysis of Shikimic Acid and Chlorophyll Concentrations

The determination of shikimic acid in maize leaves followed the procedure described by Singh and Shaner (1998), with some modifications. Frozen leaf tissue was rapidly homogenized in 0.25 M HCl, at a ratio of 0.1 g: 1.5 mL. The extract homogenate was centrifuged at 15,000 × g for 5 min at 4°C. An aliquot (200 μL) of the supernatant from the test sample was mixed with 2 mL 1% (v/v) periodic acid solution to oxidize shikimic acid. After 3 h of incubation at room temperature, the sample was mixed with 2 mL 1 M NaOH, and 1.2 mL 0.1 M glycine. The solution was thoroughly mixed, and the optical density at 380 nm was measured immediately to quantify the shikimic acid concentration, following reference to a calibration curve.

For the determination of chlorophyll concentration, the pigments were extracted from 0.1 g frozen leaf tissue immersed with 2 mL of 95% ethanol for 24 h in dark environment. Chlorophyll a and b concentrations were then determined spectrophotometrically according to Arnon (1949). Shikimic acid and chlorophyll concentrations were expressed on a fresh weight basis.

### Data Analysis and Image Visualization

Chemometric methods, including partial least squares discriminant analysis (PLS-DA) (Feng et al., 2017), PLSR (Wold et al., 2008), SPA (Liu and He, 2009), and RF algorithm (Li et al., 2012) were used in the present study to investigate and screen the responses of the WT and TG maize plants to glyphosate stress.

Hyperspectral imaging data contains redundant multicollinearity information among contiguous wavelengths (Liu et al., 2014). SPA is a forward selection approach, which selects combination of variables with minimal collinearity information (Wang et al., 2015). The principle of the SPA method was described by Liu and He (2009). In this case, SPA was firstly used to select sensitive wavelengths and to speed up the prediction models from hyperspectral imaging data. The sensitive bands could be determined on the basis of the smallest root mean error of prediction in validation set of multiple linear regression calibration (Liu and He, 2009). The RF approach works on an iterative pattern. This selection method is based on the reversible-jump Markov chain Monte Carlo, and its output features provide the relative selection probability, identifying the important feature (Li et al., 2012). In the present work, RF was performed on all the ChlF parameters to extract the important photosynthetic fingerprint for detecting the response to glyphosate treatment of the crop canopy.

Partial least squares is a powerful chemometric method which has proved to be stable, accurate and highly predictive (Wold et al., 2008). It can handle both univariate and multivariate responses and is computationally fast. Leave-one-out cross-validation was used to determine the optimal number of latent variables (LVs) in the calibration model. PLSR is used to find the fundamental relations between two matrices (X and Y) and to explore the linear regression model between X and Y. For spectral data, PLSR was used to implement predictive modes based on the full wavelengths and the sensitive wavelengths. The X matrices presented the spectral data, and the Y matrices presented the shikimic acid concentrations. For ChlF parameters, the PLSR model was developed to construct the predictive models based on the ChlF parameters and ChlF features. The two matrices, X (here: chlorophyll fluorescence parameters) and Y (here: shikimic acid concentrations), were interactively decomposed into LVs. Before establishing the prediction models, we detected the outliers for both spectral data and ChlF parameters based on the prediction of shikimic acid concentration by the PLSR model, using all the samples. After removing the outliers, the data set was divided into two subsets by sample set portion based on the joint x–y distances algorithm (Galvão et al., 2005), a calibration set containing 144 samples and a prediction set with the remaining 72 samples.

Partial least squares discriminant analysis was used to find the discrimination of glyphosate phytotoxicity damage groups for the purpose of classification the glyphosate-tolerant transgenic plants. For both spectral data and ChlF parameters, the PLS-DA model was established by assigning a dummy variable 1 or 2 reference values for all the sample. It is an arbitrary number that indicates whether the sample belongs to a specific group or not. The health samples included all the TG plants and WT plants sprayed with water, while samples with glyphosate phytotoxicity damage included those of WT plants sprayed with glyphosate. The health plants were assigned a value of 1, and those glyphosate phytotoxicity damage plants were assigned a value of 2. It was health sample if the value was between 0.5 and 1.5. The glyphosate phytotoxicity damage sample would be classified correctly if the values was between 1.5 and 2.5, otherwise, the sample was considered as incorrectly. The data set of every treatment time was divided into two subsets with the ratio of 3:1, a calibration set containing 45 samples and a prediction set with the remaining 15 samples. The accuracy of the discrimination for both calibration and prediction sets is expressed as the fraction of correctly classified samples to the total samples.

Each pixel in a hyperspectral image has a corresponding spectral fingerprint which provides the foundation for constructing the chemical images (Zhang et al., 2016a,b). Regions of the image with the same spectral information should have the same chemical composition. It was impossible to measure the physiological parameters of each pixel, so the average value for the physiological parameters and the corresponding spectrum from each sample were used for calibration. To develop a low-cost multispectral imaging device for further plant breeding application, the determination of the corresponding chemical composition of each pixel on the hyperspectral images was always achieved with the calibration model established with the sensitive wavelengths. In the current study, the optimal prediction model, combined with the image processing method, was used to establish the shikimic acid concentration prediction map for visualizing the spatial distribution between the samples.

**FIGURE 1:**
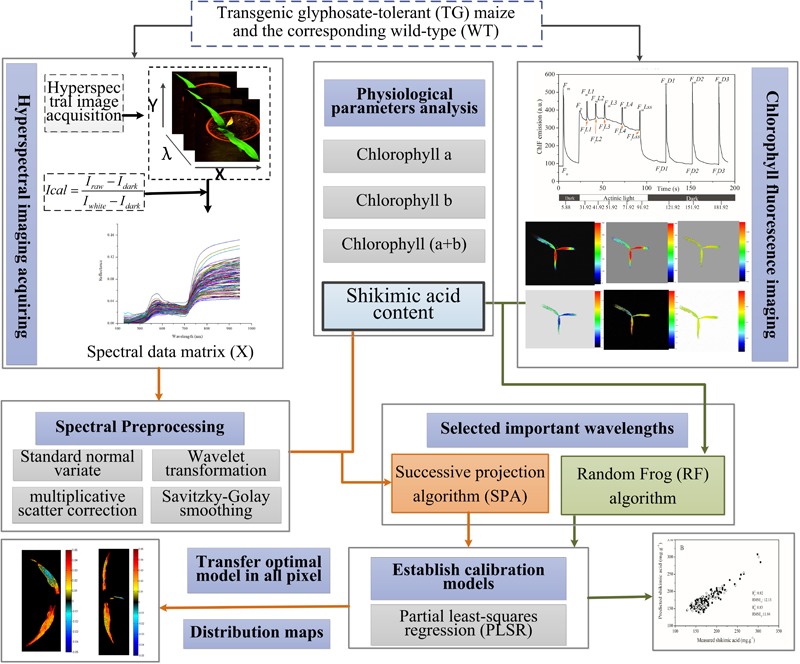
Flowchart of determination of shikimic acid concentration in maize leaves based on chlorophyll fluorescence and hyperspectral imaging.

The performances of the PLSR models were evaluated by calculating the coefficient of determination for the calibration sets (*R*2 c) and the prediction sets (*R*2 p), as well as the root mean square error of the calibration (*RMSE_C_*) and prediction (*RMSE_P_*) sets. A better model should have a higher coefficient of determination and lower root mean square error value for both calibration and prediction sets. **Figure 1** shows the main steps for the whole procedure.

### Statistical Analysis

Statistical comparisons were made by a one-way analysis of variance (ANOVA). The differences between means were established using the Student–Newman–Keuls tests (*p* < 0.05) by SPSST version 18.0 statistical program (SPSS Inc., Chicago, IL, United States). Unscrambler X version 10.1 software (CAMO AS, Oslo, Norway) was used to process the PLSR and PCA programs. The SPA and RF methods were developed using the relative toolbox of MATLAB R2014b (The MathWorks, Natick, MA, United States). The chemical image was also developed in MATLAB R2014b. OriginPro 9.0SR0 (Origin Lab Corporation, Northampton, MA, United States) graphing software was used to draw the graphs.

## Results

### Comparison of Glyphosate Injury Levels

**FIGURE 2:**
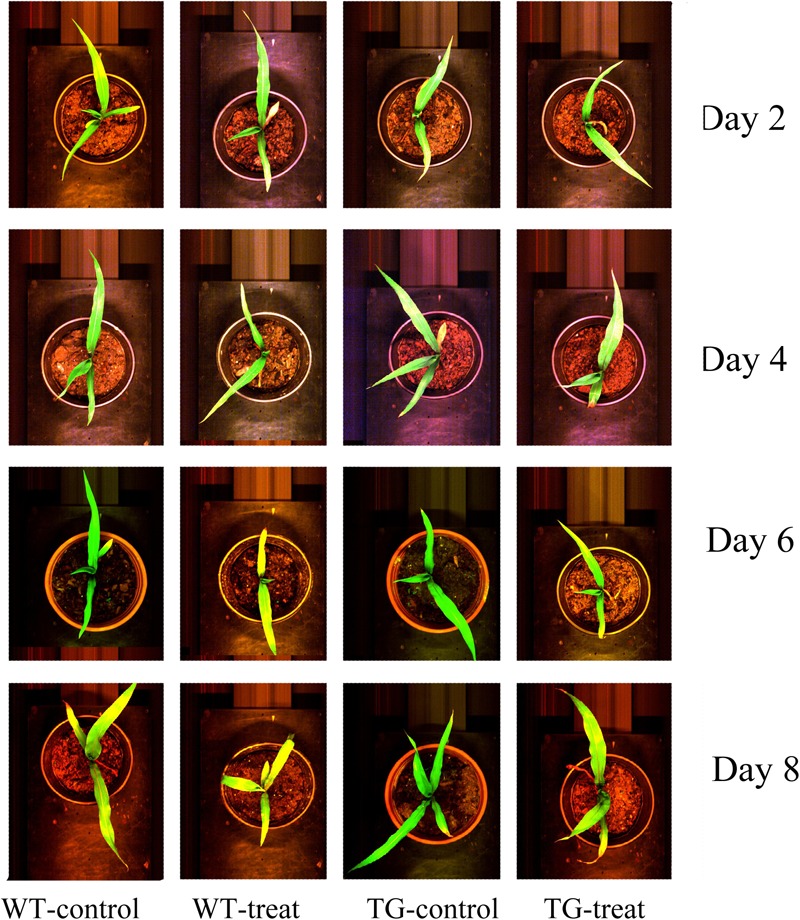
Phenotype of wild-type (WT) and transgenic (TG) maize sprayed with glyphosate after 2, 4, 6, and 8 days, compared with the corresponding control ones (in their original RGB color format).

In our research, the glyphosate-tolerant maize line, SK12-5, was obtained by expression of the bacterial EPSPS from *Agrobacterium* sp. strain CP4. This TG maize is highly tolerant to glyphosate and showed no visible injury 8 days after spraying with glyphosate (**Figure 2**).

**FIGURE 3:**
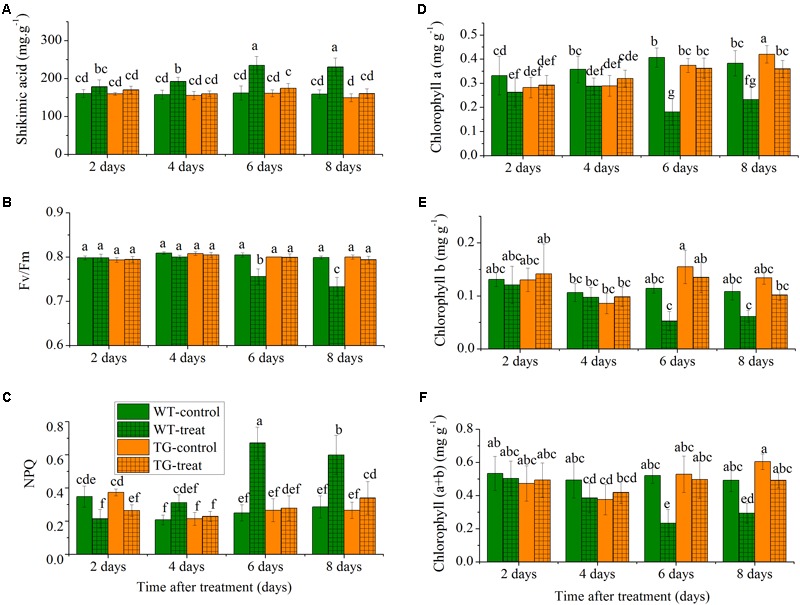
Time-course effect of glyphosate on the responses of leaf shikimic acid concentration **(A)**, *F*_v_*/F*_m_ **(B)**, *NPQ* **(C)**, chlorophyll a **(D)**, chlorophyll b **(E)**, and chlorophyll (a+b) content **(F)** in WT and TG maize plants. Values are presented as the means plus error bars, where the error bars represent the SD of the mean. Within a graph, any two bars with a common letter are not statistically significant (*P* > 0.05).

The levels of shikimate in a damaged crop indicate glyphosate injury as glyphosate inhibits the biosynthesis of aromatic amino acid biosynthesis, which causes accumulation of the precursor, shikimic acid, in plants (Singh and Shaner, 1998). The concentration of shikimic acid increased rapidly with time in the glyphosate-treated WT maize, but, as expected, low levels of shikimate were observed in TG maize plants compared to WT after the same number of days following treatment with glyphosate (**Figure 3A**). The shikimic acid concentration increased in WT maize over the time course but remained relatively constant for TG maize. For example, the amount of shikimic acid in WT maize was 1.4 times more than in TG plants at 8 days.

To further investigate the relative response of the two maize genotypes, we compared the effect of glyphosate stress on the time course of the response of two common ChlF parameters, *F*_v_*/F*_m_ and *NPQ*. The parameter *F*_v_*/F*_m_ reflects the photochemical efficiency of PSII and is used as a sensitive bioindicator to evaluate a plant’s photosynthetic performance (Porcarcastell et al., 2014), while *NPQ* reflects heat-dissipation of chlorophyll excitation energy in antenna systems and is considered to be a good indicator of “excess excitation energy” (Maxwell and Johnson, 2000). We observed clear differences in glyphosate responses between WT and TG maize. The results demonstrated that glyphosate produced phytotoxic effects on WT maize and caused significant decreases in photosynthesis, with decreased *F*_v_*/F*_m_ and increased *NPQ* 6 days after glyphosate application (**Figures 3B,C**). On the other hand, *F*_v_*/F*_m_ and *NPQ* were unchanged for TG maize during the experimental period after glyphosate application.

Additionally, the leaves of WT appeared both chlorotic and necrotic after 8 days exposure to glyphosate. The concentrations of the different chlorophylls (chlorophyll a, chlorophyll b, and chlorophyll a+b) decreased in the leaves of the WT in response to glyphosate, whereas, in the case of TG, there was no significant effect of glyphosate treatment on chlorophyll concentrations (**Figures 3D–F**).

### Time Course of Hyperspectral Spectral Data Response to Glyphosate Treatment

**FIGURE 4:**
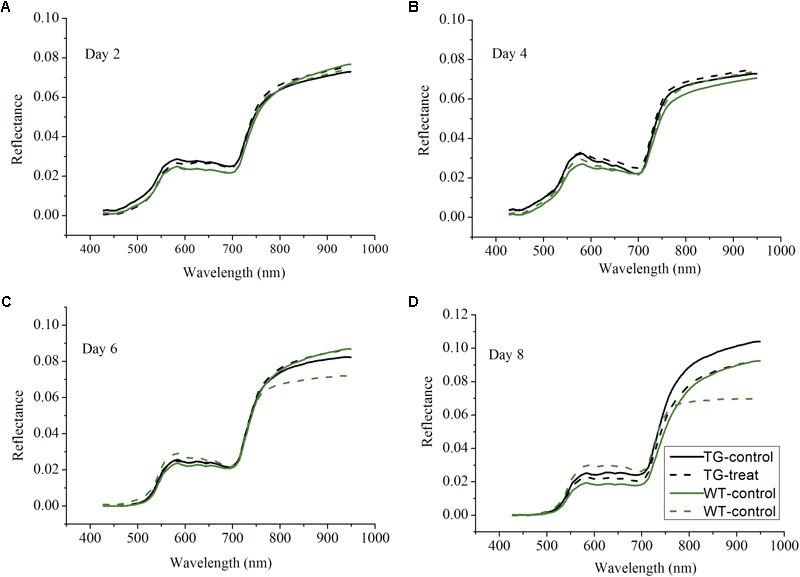
Profiles of average spectra from the visible and near-infrared ranges after 2 **(A)**, 4 **(B)**, 6 **(C)**, 8 **(D)** days glyphosate treatment on TG and WT maize plants.

Following on from the results which showing that the destructive assays to determine shikimic acid and chlorophyll concentrations demonstrated differences between the response to glyphosate of the WT and TG genotypes, non-invasive techniques, namely Vis-NIR HIS and ChlF imaging, were then tested to detect effects on photosynthesis. The Vis-NIR spectral dataset consisted of 216 samples, including TG and WT maize plants, over the spectral range of 380.67–1030.03 nm. The beginning and end regions of the spectra were accompanied by a large amount of noise caused by the optical instrument and the measurement conditions, so were excluded and only the range of 427.75–948.49 nm was used for analysis. The Vis-NIR spectral characteristics of the plant canopy showed important information related to the pigment concentrations, cell structures, and the biochemical composition of the leaves (Rathod et al., 2013). All the sample spectral profiles showed similar spectral trends, but there were obvious average spectral reflectance value differences between the genotypes (TG vs. WT) and the treatments (water vs. glyphosate) 6 days after glyphosate (or water) application (**Figure 4**). WT plants treated with glyphosate exhibited different spectral behavior than did either glyphosate-treated TG plants or the WT control several days after treatment. In the visible region of 400–780 nm, WT plants treated with glyphosate had higher spectral reflectance values than did glyphosate-treated TG plants and WT control plants after 6 days treatment, which might be caused by the decrease in chlorophyll concentration. In the NIR region (780–950 nm), glyphosate-treated WT plants had lower reflectance values 6 days after glyphosate treatment than did glyphosate-treated TG and control WT plants, effects which were mainly caused by influences on biochemical composition and cellular structure. Glyphosate is a slow-acting herbicide that takes a long time to exhibit the visual symptoms of herbicide damage (Sammons and Gaines, 2014; **Figure 2**). Therefore, the phenotyping difference in response to glyphosate stress between the two maize genotypes (tolerant TG and sensitive WT) was captured by the Vis-NIR spectral signature after 6 days treatment.

**FIGURE 5:**
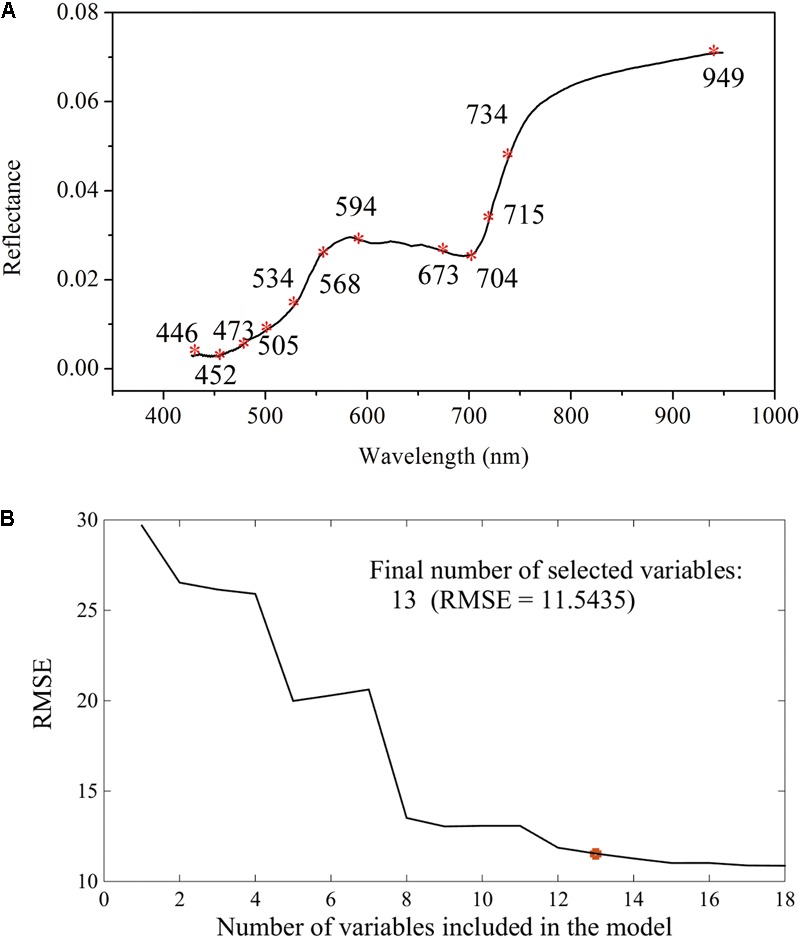
Selection of sensitive wavelengths by the successive projections algorithm: distributions of important variables for shikimic acid concentration **(A)**; final number of selected variables determined on the basis of the root mean square error (RMSE) of validation set of multiple linear regression models **(B)**.

The canopy Vis-NIR spectral signal response to gene-environment interactions is the theoretical foundation for finding the mathematical relationships between spectral information and the glyphosate-induced effects. The amount of shikimate accumulation in response to glyphosate treatment is the quantitative index for examining glyphosate tolerance of a plant. Therefore, the combination of spectral information and multivariate analysis using PLSR was applied to achieve rapid and real-time quantification of shikimate concentrations in different genotypes of maize under stress and control environments. It is universally known that the Vis-NIR HIS dataset has high dimensionality of variables that always exhibit collinearity (Liu et al., 2014). Recent research has proposed band selection using improved sparse subspace clustering (Sun et al., 2015), dissimilarity-weighted sparse self-representation (Sun et al., 2016), SPA, uninformative variable elimination, competitive adaptive reweighted sampling (Zhang et al., 2016a), and so on. Here, SPA was firstly implemented to select the sensitive wavelengths which carried the most significant information for predicting shikimic acid concentration. Thirteen wavelengths (446, 452, 473, 505, 524, 534, 568, 594, 673, 704, 715, 734, and 949 nm), with RMSE of 11.54, were identified by the SPA method (**Figure 5**). Optimal wavelengths selection aims at selecting wavelengths subset has maximum information and the selected appropriate wavelengths subset with minimum correlation with other wavelengths (Sun and Liu, 2012). Therefore, simple correlation analysis was conducted between each selected wavelengths and their spectral response to corresponding shikimic acid concentration (Supplementary Figure S2A). There were still high correlation coefficients found between some wavelengths. PLSR model was implemented to evaluate the actual roles of sensitive wavelengths. The predictive capability of PLSR model was reduced with the input number of wavelengths decreased (Supplementary Figure S2B). When the number of wavelengths was decreased to 11, the PLSR model was still considered as acceptable with *R*^2^_c_ of 0.79 (*RMSE_C_*, 13.36) and *R*^2^_p_ of 0.82 (*RMSE_P_*, 13.38). Therefore, 11 wavelengths were selected as the sensitive wavelengths related to shikimic acid concentration. The majority of the sensitive wavelengths were located in the Vis region (380–780 nm) as a result of light absorption by the chlorophylls (Pan et al., 2015), which is consistent with the results that chlorophyll concentration decreased in glyphosate-treated WT plants (**Figures 3D–F**). In addition, the absorptions at ∼950 nm were assigned to the second overtones of O-H stretching of water (Zhao et al., 2016).

**FIGURE 6:**
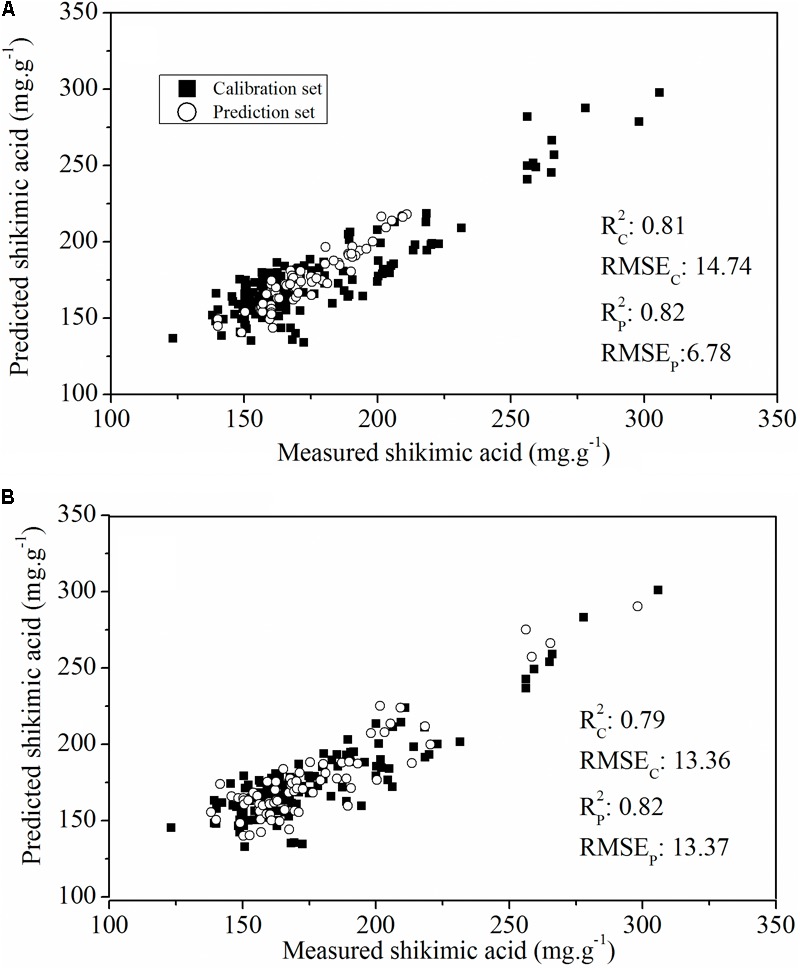
Plot of reference vs. predicted values of shikimic acid concentration derived from the PLSR model based on the whole spectrum **(A)** or the sensitive wavelengths **(B)**.

To evaluate the actual roles of individual features, the full spectral range (412 wavelengths) and the above-mentioned sensitive wavelengths (11 wavelengths) were therefore set as the input of the PLSR model (**Figure 6**). The PLSR model established over the whole range of Vis-NIR spectral data fitted well with the results for shikimic acid concentration, with an *R*2 c value of 0.81 and an *R*2 p value of 0.82 (**Figure 6A**). The optimal PLSR model was obtained with nine LVs. When the number of wavelengths was decreased to 2.67% of full spectral range, the PLSR model developed for the sensitive wavelengths had similar statistical characteristics compared to that based on the whole spectrum (**Figure 6B**). The first nine LVs were selected to establish the optimal model. The optimal model for constructing the quantitative relationship between shikimic acid concentration and spectral reflectance is shown as follows:

$$\begin{array}{c} Y\;=\;\text{484}.{\text{65X}}_{\text{704}}{\;}_{\text{nm}}\text{ − 1018}.{\text{89X}}_{\text{5343}}{\;}_{\text{nm}}\text{ − 887}.{\text{77X}}_{\text{673}}{\;}_{\text{nm}}\text{+ }\\ \;304.06X_{568}{\;}_{\text{nm}}+1645.31X_{505}{\;}_{\text{nm}}+354.86X_{734}{\;}_{\text{nm}}+\\ \;1092.13X_{524}{\;}_{\text{nm}}+449.62X_{949}{\;}_{\text{nm}}-1997.31X_{446}{\;}_{\text{nm}}\\ -589.93X_{594}{\;}_{\text{nm}}+1283.30X_{452}{\;}_{\text{nm}}-1126.19\; \end{array}$$

where *Y* is the predicted shikimic acid concentration, and Xi nm is the sensitive wavelength of the reflectance spectra.

**FIGURE 7:**
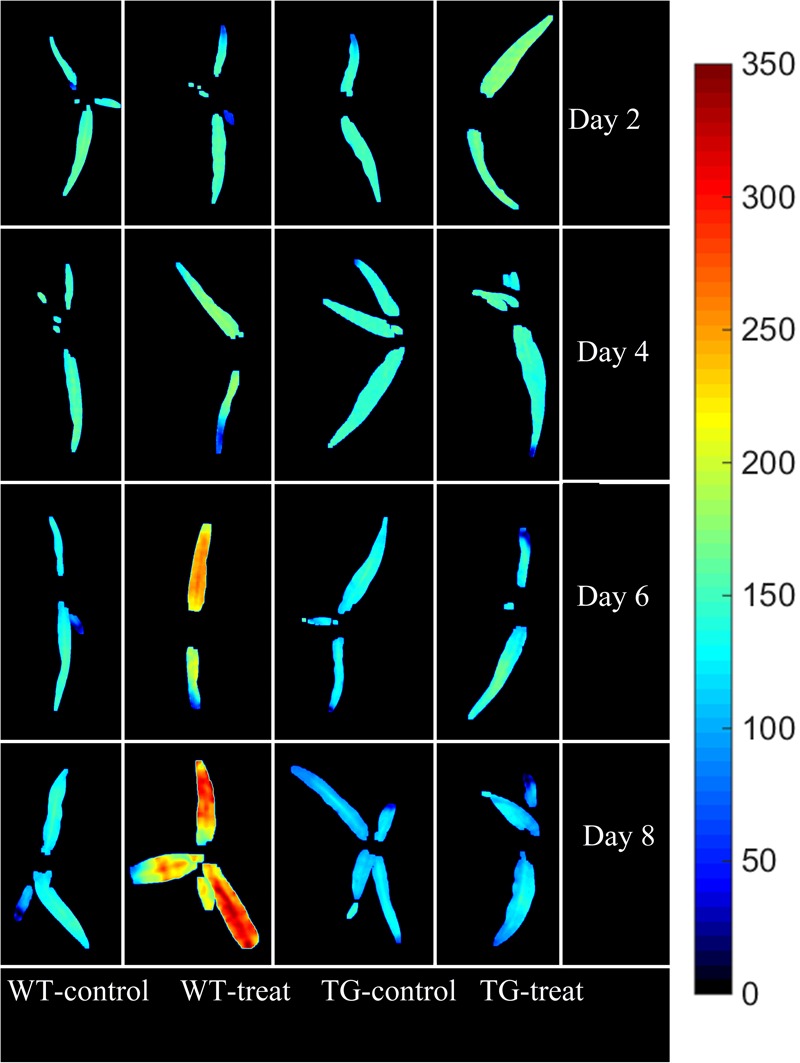
Visualization of the response (over time) of shikimic acid concentration to glyphosate treatment in WT and TG maize. The color bar, from blue to red, shows increasing levels of shikimic acid concentration.

The final step in employing hyperspectral imaging was to construct the chemical image for rapid and non-invasive detection of plant phenotyping changes caused by glyphosate stress. On the basis of the established calibration model, the shikimic acid concentration of every pixel in the examined hyperspectral images can be obtained by transferring the optimal model to each pixel. **Figure 7** illustrates the visualized image of the response over time of shikimic acid concentration in maize to treatment with glyphosate. There were no obvious morphological changes between TG and WT under glyphosate treatment at the early stages. With HIS, however, the differences between WT and TG plants were obvious, evolving from warm colors (high shikimic acid values) to cold colors (low shikimic acid values) after 6 days of treatment.

### Time Course of Changes in Chlorophyll Fluorescence Characters in Response to Glyphosate Treatment

**FIGURE 8:**
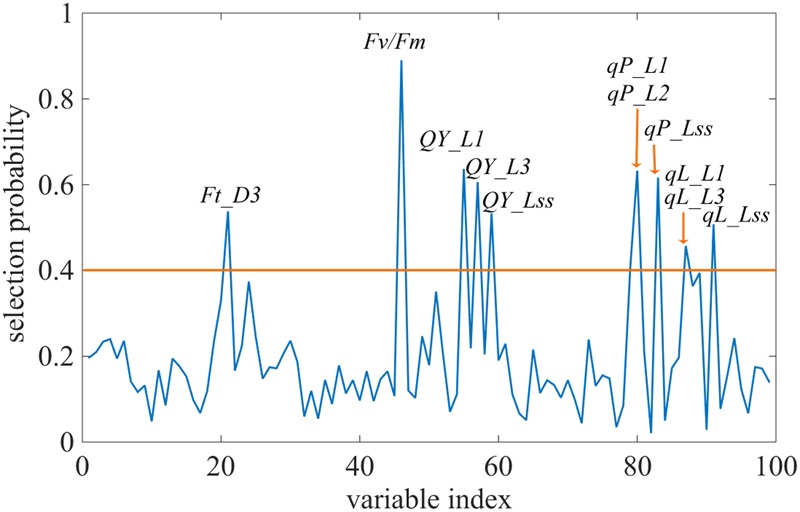
Selection probability of each feature by the Random Frog algorithm. The orange line highlights the selection probability threshold of 0.4.

**FIGURE 9:**
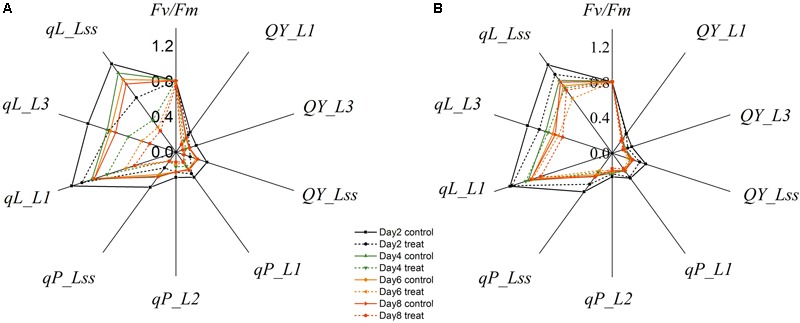
Relative changes in mean values of selected chlorophyll fluorescence parameters in WT **(A)** and TG **(B)** maize plants treated with glyphosate.

Changes in the commonly used ChlF parameters, *F*_v_*/F*_m_ and *NPQ*, indicated that glyphosate supply could cause changes in the physiological state of the photosynthetic machinery of the WT plants, and could be used to distinguish glyphosate-tolerant from glyphosate-sensitive genotypes (**Figures 3B,C**). However, they might be not the best biomarkers for identifying superior plant phenotypes. We used the RF feature selection method to identify parameters that showed the most sensitive response (**Figure 8**). In the present study, a small number of ChlF parameters displayed with high selection probabilities (over 0.4). Most of the parameters were with low selection probabilities. For the first 20 significant ChlF parameters selected by RF, the selection probabilities were 0.918, 0.673, 0.671, 0.666, 0.614, 0.581, 0.547, 0.530, 0.486, 0.451, 0.411, 0.389, 0.377, 0.372, 0.356, 0.308, 0.294, 0.273, 0.272, and 0.266, respectively. PLSR model was implemented to evaluate the actual roles of individual features selected by RF. Supplementary Figure S3 shown the result of coefficient of determination by number of ChlF parameter included in the model. *R^2^_c_* value increased with the number of parameter in the model. However, the *R^2^_c_* did not increased when the number was eleven. In the present study, eleven ChlF parameter displayed selection probabilities larger than 0.4. Therefore, the ChlF parameters with selection probabilities higher than the threshold of 0.4 were selected as features. And their performances were related to the response of WT and TG maize to glyphosate treatment (**Figure 9**). The distance from the center of the spider plot indicated the relative change in the selected ChlF parameters over the entire evaluation period. We observed clear differences in these features in response to glyphosate stress between WT and TG maize. No symptoms were visible in WT until 8 days after application of glyphosate, when the leaves of maize became withered and necrotic. In contrast, no such symptoms were evident in glyphosate-treated TG maize at any point during the experimental period. The foliar application of glyphosate impaired the PS II photochemical efficiency of the WT plants, which was clearly reflected in the significantly reduced values for *F*_v_*/F*_m_, *qL, Qp*, and *QY* in the first few days after glyphosate application (**Figure 9A**). For TG maize plants, the selected ChlF features recorded in glyphosate-treated plants remained similar to those recorded in the control WT plants (**Figure 9B**).

**FIGURE 10:**
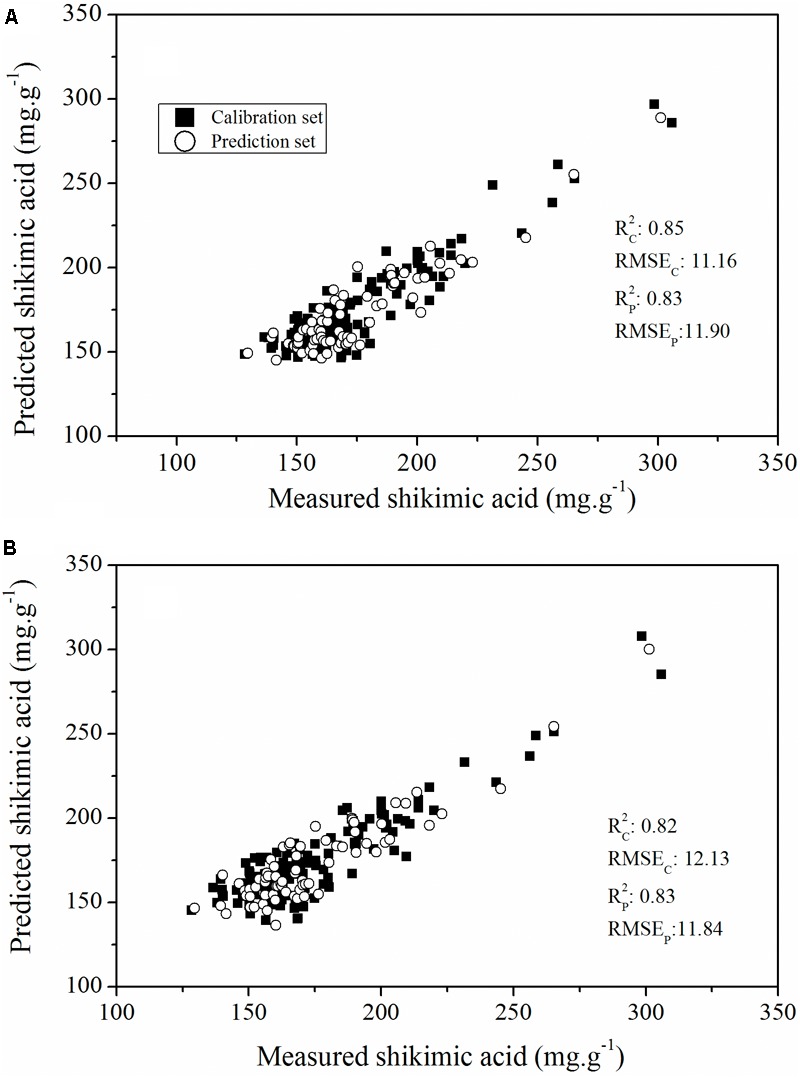
Plot of reference vs. predicted values of shikimic acid concentration, derived from the PLSR model based on all the ChlF parameters **(A)** and the sensitive features **(B)**.

To further assess the capability of features selected using the RF approach to evaluate glyphosate tolerance in plant breeding populations, we developed a PLSR model based on all the ChlF parameters and glyphosate-sensitive parameters to calculate the relationship between the ChlF parameters and glyphosate stress-induced shikimic acid concentration (**Figure 10**). The optimal number of LVs in the PLSR models were all defined as three. The PLSR model established that all the ChlF parameters had a better predictive capability than the model established on the glyphosate-sensitive features, with an *R*_c_^2^ value of 0.85 (*RMSE_C_* = 11.16) and an *R*_p_^2^ value of 0.83 (*RMSE_P_* = 11.90). The PLSR model calculated on parameters for shikimic acid concentrations in leaves was less accurate, but was still considered acceptable (*R*_c_^2^, *RMSE_C_, R*_p_^2^, *RMSE_P_* were 0.82, 12.13, 0.83, and 11.84, respectively). These results demonstrated the capability of the selected ChlF parameters to be optimal candidates for fluorescence phenotyping markers in transgenic maize glyphosate-tolerance breeding programs.

**FIGURE 11:**
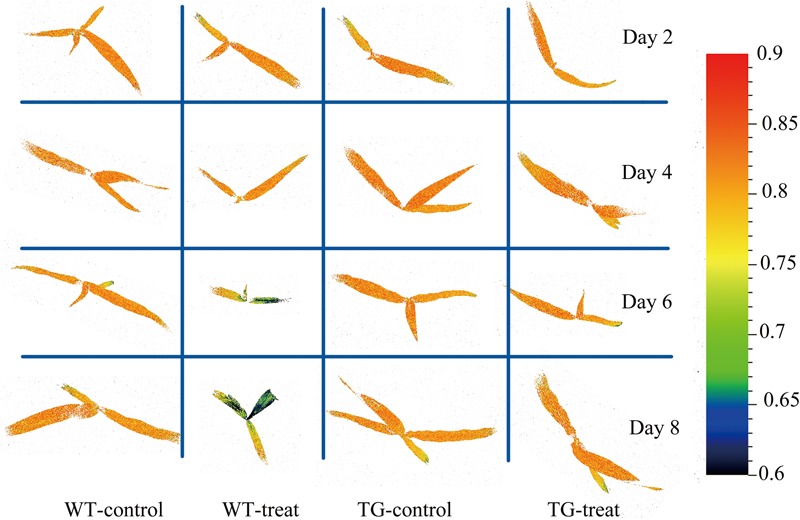
Chlorophyll fluorescence images of maximum photochemical quantum yield of PSII (*F*_v_*/F*_m_) for the whole canopy of WT and TG maize during the time course study of response to glyphosate application. The color bar on the right side of the image represents the ranges of values and how they mapped to the color palette.

Chlorophyll fluorescence images showed heterogeneities and temporal variations over the whole plant. Local changes in *F*_v_*/F*_m_ (the most glyphosate-sensitive parameter selected by the RF method) were observed between WT and TG maize (**Figure 11**). The given image was plotted on a false color pattern, where the corresponding color represents its relative value to the fluorescence intensity of the pixel. As expected, *F*_v_*/F*_m_ decreased during the time-course of response to glyphosate in WT maize. However, this damage to PSII (suggested by *F*_v_*/F*_m_) seems to be a late event in the present research. The first pre-symptomatic effects were detected at 6 days after glyphosate application to WT maize, using images of the ChlF parameter *F*_v_*/F*_m_. Effects like the decrease of *F*_v_*/F*_m_, the increase in *NPQ* and the concomitant reduction of *qL, qP, QY* are bioindicators for evaluating the effects of induced damage on the photosynthetic machinery.

### Classification of Glyphosate-Tolerant Maize

Once it was determined that spectral information and ChlF fingerprint could be used for predicting shikimic acid concentration, we tested the approach by establishing a model capable of discriminating the glyphosate-tolerant TG maize. The classification model were conducted on the sensitive wavelength and significant ChlF parameters. **Table 1** shows recognition accuracies obtained from the calibration and prediction sets. For the first few days after treatment, the classification ability of PLS-DA model calculated on spectral information was slightly worse than that obtained from ChlF information. The calibration set was 97.78% accurate and 93.33% for prediction set 6 days after spraying with glyphosate based on ChlF parameters. The classification capacity of PLS-DA models established on spectral features were also acceptable, with accuracy of 91.11% for calibration set. The PLS-DA established on spectral information and ChlF parameter achieved same recognition results at 8 days application of glyphosate. The result demonstrated that glyphosate-tolerant transgenic maize could be identified 6 days after glyphosate treatment with the developed protocol.

**Table 1:** Performance of the discrimination models based on the sensitive wavelengths and sensitive chlorophyll fluorescence parameters.

Time after glyphosate treatment	Sensitive wavelengths			Sensitive chlorophyll fluorescence parameters		
	Par^[a]^	Calibration set	Prediction set	Par^[a]^	Calibration set	Prediction set
2 days	9	88.89%	68.89%	2	84.44%	80.00%
4 days	6	88.89%	80.00%	3	91.11%	80.00%
6 days	8	91.11%	80.00%	6	97.78%	93.33%
8 days	10	95.56%	100%	2	95.56%	100%

*^*[a]*^Model parameters indicate the optimal number of latent variables for establishing the partial least squares discriminant analysis calibration model.*

## Discussion

Glyphosate is currently one of the most important active ingredients for weed control, being toxicologically and environmentally safe, as well as offering use as a growth regulator (Duke, 2008). However, glyphosate is a broad-spectrum herbicide that also injures or kills following post-emergence application to crops, when it is in direct contact with foliage. The introduction of transgenic glyphosate-tolerant crops, associated with post-emergence glyphosate use, has resulted in a major reduction in the use of other herbicides (Shaner, 2000). TG maize, transformed with a bacterial gene encoding a glyphosate-tolerant EPSPS, showed more than 100-fold greater tolerance to glyphosate in comparison with its parental control at the third-leaf stage (Hetherington et al., 1999).

An important procedure in plant breeding is the fixation of superior plant phenotypes in the development of improved cultivars suited to crop breeders. All mentioned characteristics evaluated, including shikimate acid concentration, ChlF parameters, chlorophylls, indicted that glyphosate treatment resulted in photosynthetic efficiency reduction and photosynthetic apparatus damage of WT maize (**Figure 3**). TG maize showed substantially greater resistance to glyphosate application than the corresponding untransformed parental control. However, the biochemical analysis methods for measuring shikimate acid concentration and chlorophyll concentration are always destructive, time-consuming, and having tedious and high costs; thus, it is unsuitable for online application. Therefore, Non-invasive and high-throughput imaging techniques, including Vis-NIR HIS and ChlF imaging, were applied to plant phenotyping, and the results analyzed and compared in the next step of the research.

Sub-lethal concentrations of glyphosate had a significant effect on the leaf water content of *Bolboschoenus maritimus* after prolonged treatment (Mateos-Naranjo and Perez-Martin, 2013). It is suggested that glyphosate might alter the internal cellular structure and physiology, resulting in changes in chlorophyll concentration and leaf water content, which was consistent with a previous study (Gomes et al., 2014). In the present study, Vis-NIR HIS combined with chemometric methods was assessed to predict shikimic acid concentration in leaves. A calibration model was successfully developed by PLSR, based on 11 sensitive wavelengths, with high *R*^2^-values of 0.79 for the calibration set and 0.82 for the prediction set (**Figure 6B**). In addition, glyphosate injury was shown clearly in the shikimic acid concentration prediction map (**Figure 7**). The results indicate that phenotyping system established by HIS cameras can be used to screen the glyphosate tolerance crop and detect the changes of glyphosate stress for the breeding purposes. However, the low resolution of the Vis-NIR imaging system and the need for algorithms to achieve image segmentation caused partial loss of pixel information, resulting in changes in crop traits in the prediction map.

Chlorophyll fluorescence parameters have the potential to quantify glyphosate-induced stress in the first few days more effectively than do visual evaluations, and make it possible to differentiate tolerant genotypes from those sensitive to the aforementioned stress. Photosynthesis-targeting herbicides, such as diuron, are known to interrupt the PS II electron transport chain and thus reduce the ability of the plant to turn light energy to chemical energy (Rutherford and Krieger-Liszkay, 2001). Consequently, alteration of photosynthetic electron flow caused by diuron can be measured by monitoring induced ChlF parameters. Haynes et al. (2000) studied the impact of diuron on photosynthesis in seagrass by measuring ChlF. A decline in effective quantum yield was found with 2 h of diuron exposure in *Cymodocea serrulata, Halophila ovalis*, and *Zostera capricorni*. Significant changes in ChlF parameters, observed at low concentrations of diuron were also indicative of alternation in the structure of photosynthetic apparatus of *Saccharina japonica Aresch* (Kumar et al., 2010). However, glyphosate do not directly affect the photosynthetic apparatus, and in this case clear change of ChlF parameters can be detected as indirect effect of a secondary metabolic perturbation that results in damage in the photosynthetic apparatus (Hunsche et al., 2011). A clear difference in ChlF parameters, in response to glyphosate response between WT and TG maize, was observed (**Figure 9**). Glyphosate treatment of the WT regulated the light-harvesting capacity, causing the down-regulation of photochemical quenching capacity and the up-regulation of non-photochemical quenching capacity in PS II. However, this damage to PSII (suggested by the effects of *F*_v_*/F*_m_ and *NPQ*) seems to be a late event, occurring 6 days after glyphosate treatment. This is in agreement with the results reported by Mateos-Naranjo et al. (2009) for glyphosate treatments of cordgrass. The *R_c_^*2*^*(0.82), *RMSE_C_* (12.13), R_p_^*2*^ (0.83), and *RMSE_P_* (11.84) values obtained from the regression models established between the sensitive ChlF parameter and shikimic acid concentration indicated that this system could be suitable for high-throughput screening in a plant breeding program (**Figure 10**).

A decreased value of *F*_v_*/F*_m_ clearly indicated a blocked electron transport chain caused by herbicide application and subsequent damage to photosynthetic structures (Pavlović et al., 2014). The *F*_v_*/F*_m_ parameter has been used to successfully monitor the effect of glyphosate on soybean (Huang et al., 2012), willow (Gomes et al., 2016), *Bolboschoenus maritimus* (Mateos-Naranjo and Perez-Martin, 2013) and *Eleusine indica* (Zhang et al., 2015). ChlF imaging, using the parameter *F*_v_*/F*_m_, has been widely used to screen for perturbations in metabolic processes in plants exposed to abiotic and biotic stresses (Li et al., 2014). Barbagallo et al. (2003) suggested using the *F*_v_*/F*_m_ imaging to screen plants treated with the herbicide imazaphyr for evidence of metabolic perturbations. Because *F*_v_*/F*_m_ is a commonly used indicator of damage to the photochemical apparatus, we proposed that it could be the preferred parameter for screening for the glyphosate-tolerant phenotype in plant breeding programs.

Glyphosate phytotoxicity could influence photosynthetic processes, causing damage to cellular structure and interfering with leaf water and chlorophyll content, which could be captured by HIS techniques. Recording of ChlF transients and imaging in our experiments allowed the quantification of photosynthetic parameters that provide insight into the changes occurring in PSII function following glyphosate application. The multivariate chemometric analysis for predicting shikimic acid concentration and the discrimination models demonstrated that both HIS and ChlF imaging techniques could be used for screening and characterization of the glyphosate-tolerant maize genotype to facilitate plant breeding programs. In the future, more samples with a wide range of stress degree caused by glyphosate should be studied to established more accurate and robust inspection model which could be applied in plant breeding programs. Furthermore, large number of genotypes and validation of the predictions of classifications (susceptible vs. resistant) should be studied to demonstrate the potential of screening based on HIS and ChlF imaging.

## Author Contributions

XF, CY, YC, JP, LY, HW, and TS performed the measurements. XF and YH designed the experiments and wrote and reviewed the manuscript. YH reviewed the initial design of the experiments and made guidance for the writing of the manuscript. All authors reviewed the manuscript.

## Conflict of Interest Statement

The authors declare that the research was conducted in the absence of any commercial or financial relationships that could be construed as a potential conflict of interest.

## Supplementary Material

**FIGURE S1 |** Chlorophyll fluorescence trace illustrating the terminology and sequence of events for acquiring raw fluorescence images from plants. Dn, during dark relaxation; Ln, during light adaptation; Lss, in steady-state.

**FIGURE S2 |** Correlation coefficients between each selected wavelengths and their spectral response to corresponding shikimic acid concentration **(A)** and performance of PLS-DA model established on increasing sensitive wavelengths included in the model **(B)**.

**FIGURE S3 |** Results of analysis by the PLSR models for estimating shikimic acid concentration with the increasing number of sensitive ChlF parameters included in the model.

**TABLE S1 |** Prediction results of the pre-processing models constructed by partial least-squares regression (PLSR) based on spectral information for shikimic acid concentration.

## Abbreviations

*F*_m_: maximum fluorescence
*F*_v_*/F*_m_: maximum PSII quantum yield
*NPQ*: instantaneous non-photochemical quenching relaxation
PLSR: partial least-square regression
*qL*: (*F*_q_/*F*_v_)/(*F*_o_/*F*_t_); coefficient of photochemical quenching measured the fraction of open PSII centers based on a ‘lake’ model for PSII
*qP*: (*F*_m_-*F*_t_)/(*F*_m_-*F*_o_); coefficient of photochemical quenching
*QY*: (*F*_m_-*F*_t_)/*F*_m_; instantaneous PSII quantum yield
RF: random Frog
SPA: successive projections algorithm
