# Supplementary figures and images for: Non-destructive Determination of Shikimic Acid Concentration in Transgenic Maize Exhibiting Glyphosate Tolerance Using Chlorophyll Fluorescence and Hyperspectral Imaging
Source: Front Plant Sci. 2018 Apr 9;9:468. doi: 10.3389/fpls.2018.00468 (PMC5900420; doi:10.3389/fpls.2018.00468)

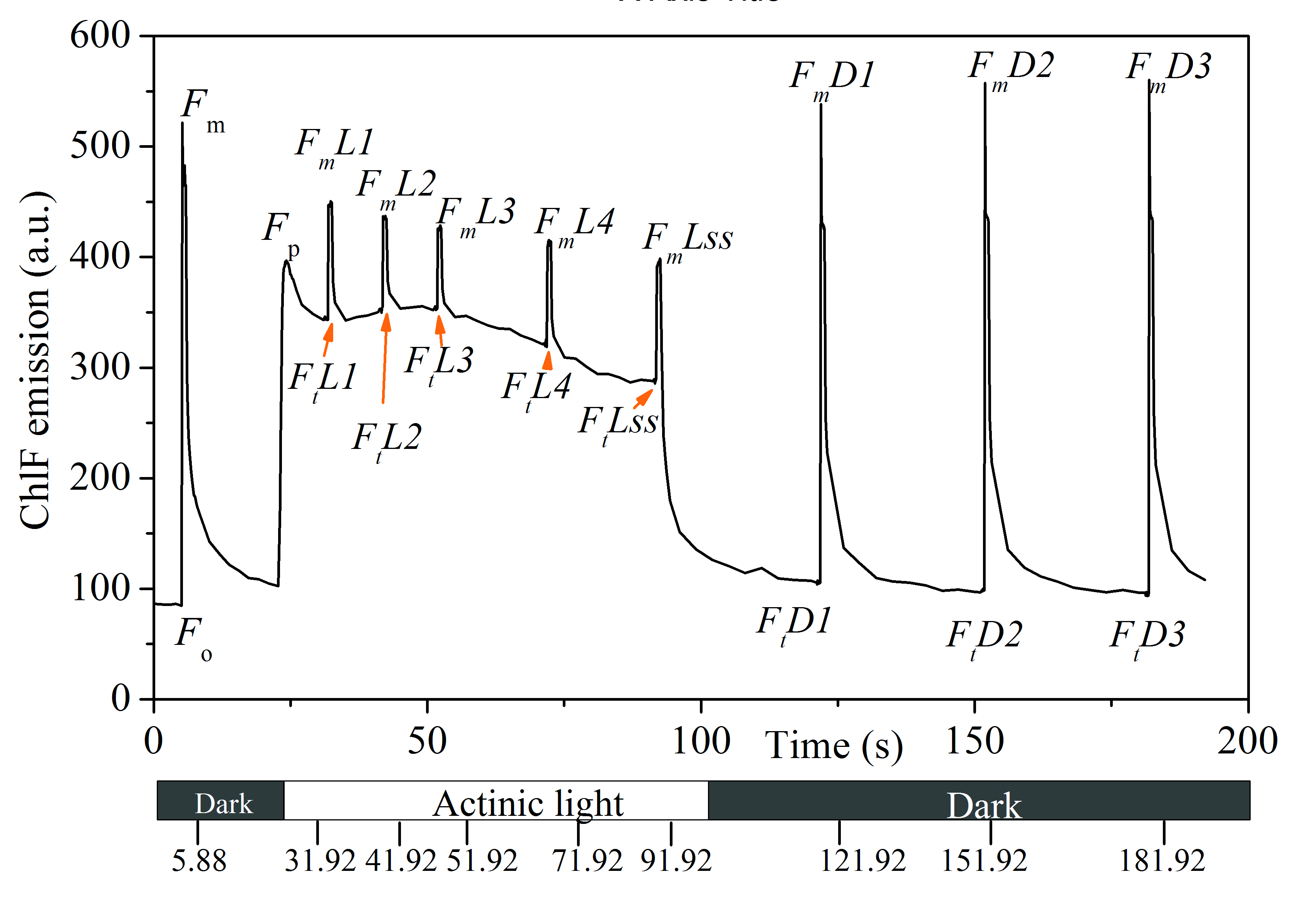

Supplement: Supplementary file 1 [file Image_1.TIF]

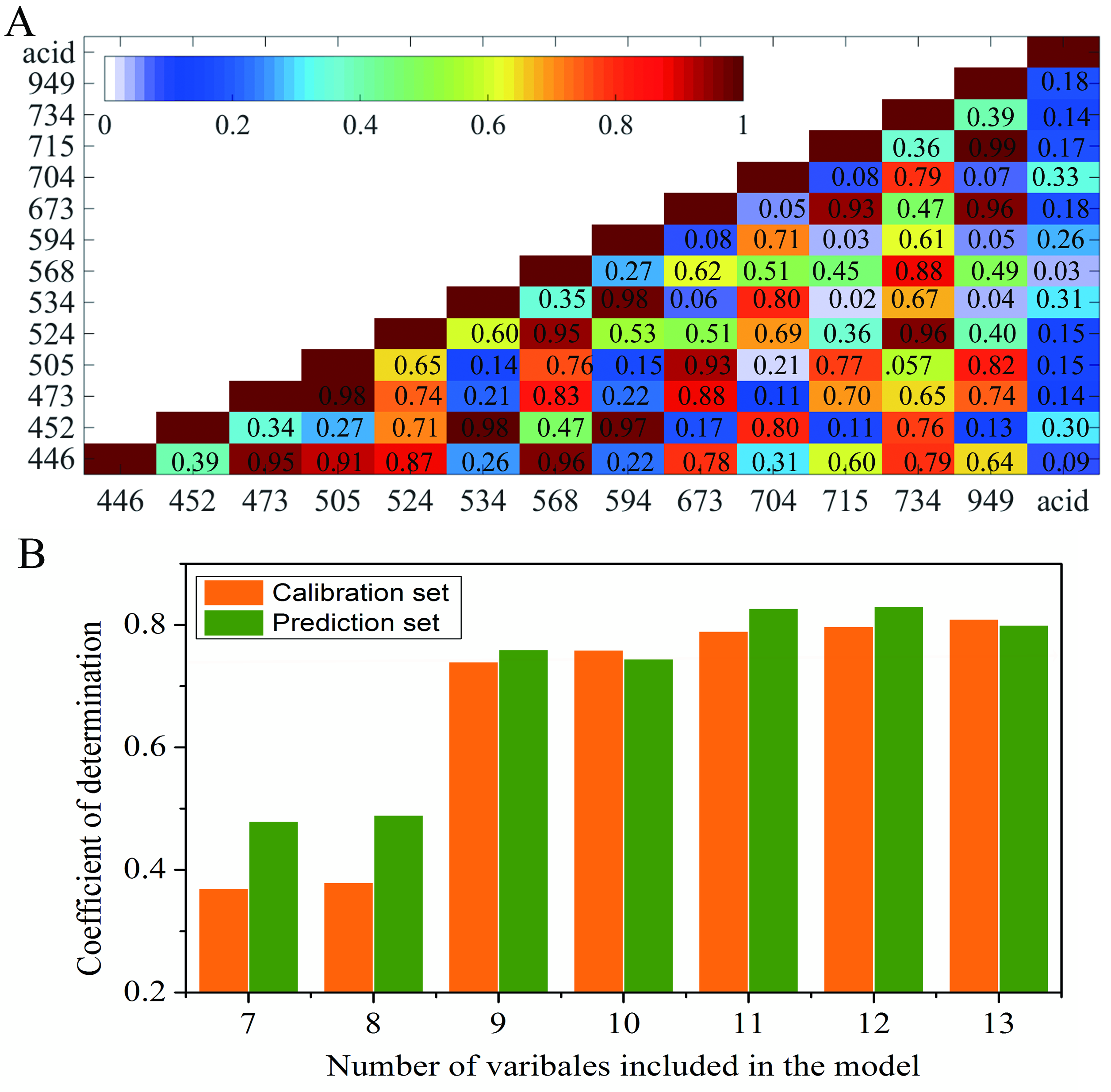

Supplement: Supplementary file 2 [file Image_2.TIF]

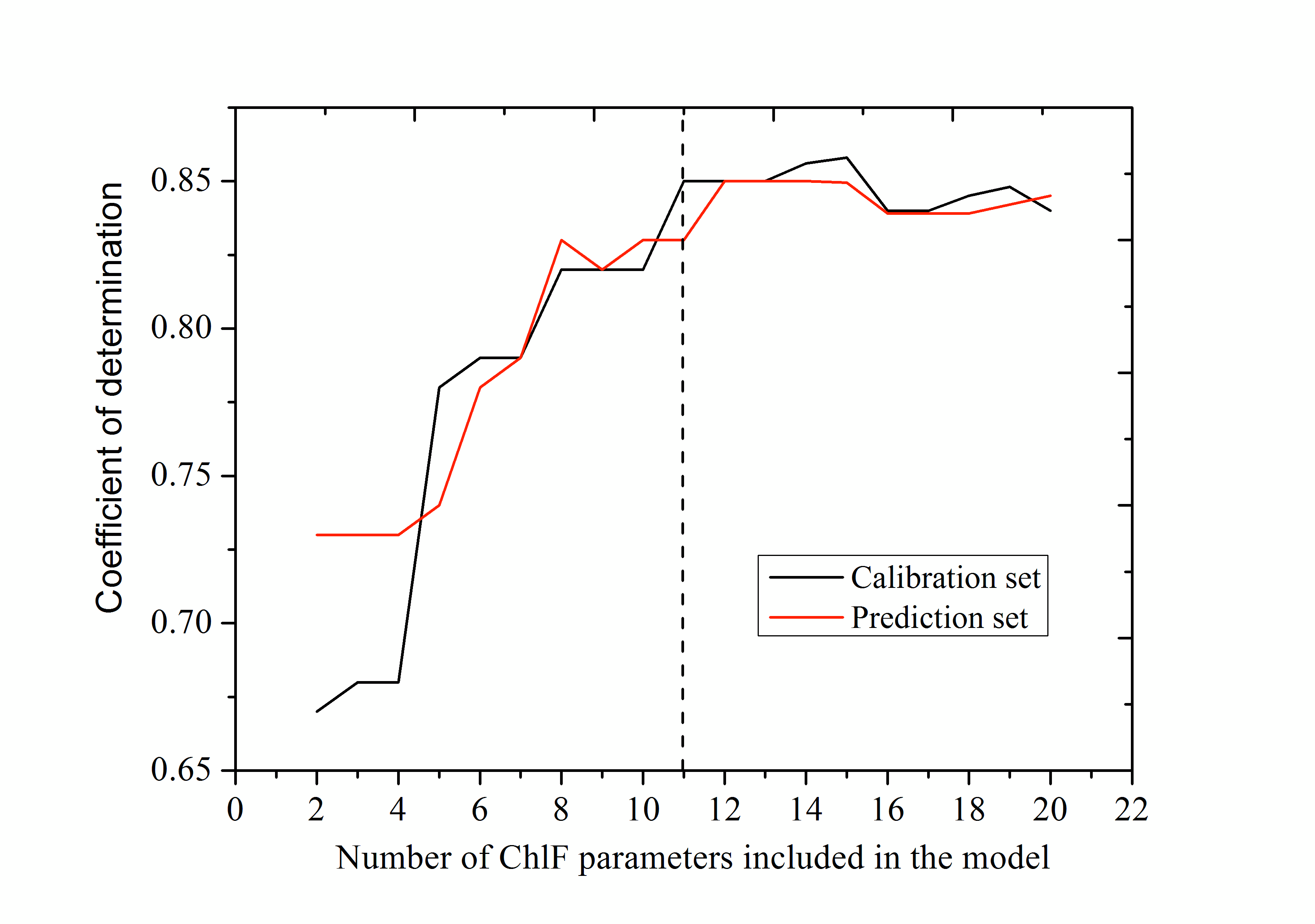

Supplement: Supplementary file 3 [file Image_3.TIF]
