# Supplementary material for: Non-destructive Determination of Shikimic Acid Concentration in Transgenic Maize Exhibiting Glyphosate Tolerance Using Chlorophyll Fluorescence and Hyperspectral Imaging
Source: Front Plant Sci. 2018 Apr 9;9:468. doi: 10.3389/fpls.2018.00468 (PMC5900420; doi:10.3389/fpls.2018.00468)
Supplement: Supplementary file 4 [file Table_1.DOCX]

Supplementary Material

Non- destructive determination of shikimic acid concentration in transgenic maize exhibiting glyphosate tolerance using chlorophyll fluorescence and hyperspectral imaging

Xuping Feng^1^, Chenliang Yu^2^, Yue Chen^3^, Jiyun Peng^1^, Lanhan Ye^1^, Tingting Shen^1^, Haiyong Wen^1^, Yong He^1^*

^1^Key Laboratory of Spectroscopy, Ministry of Agriculture, College of Biosystems Engineering and Food Science, Zhejiang University, Hangzhou 310058, China

^2^Vegetable Research Institute, Zhejiang Academy of Agricultural Sciences, Hangzhou 310021, China

^3^Institute of horticulture, Zhejiang academy of agricultural science, Hangzhou 310021, China

*** Correspondence:** Yong He: yhe@zju.edu.cn

**Supplementary Table 1.** Prediction results of the pre-processing models constructed by partial least-squares regression (PLSR) based on spectral information for shikimic acid concentration

| Model type | Par^[a]^ | Calibration set | | Prediction set | |
| --- | --- | --- | --- | --- | --- |
|  |  | *R*2 c | *RMSE_C_* | *R*2 p | *RMSE_P_* |
| MSC–PLSR | 7 | 0.73 | 15.24 | 0.73 | 15.09 |
| WT–PLSR | 9 | 0.81 | 14.74 | 0.82 | 6.78 |
| SG–PLSR | 8 | 0.73 | 14.10 | 0.76 | 14.66 |
| SNV–PLSR | 10 | 0.80 | 12.75 | 0.80 | 12.21 |

^[a]^Model parameters indicate the optimal number of latent variables for establishing the PLSR calibration model; *R*2 c and *R*2 p, coefficients of determination for calibration and prediction sets, respectively; *RMSE_C_* and *RMSE_P_*, root mean square errors of calibration and prediction sets, respectively; SNV, standard normal variate; SG, Savitzky-Golay smoothing; MSC, multiplicative scatter correction; WT, wavelet transformation
